# Features of the Structural Design of Welded Joints of Superelastic Nitinol Wires

**DOI:** 10.3390/ma19010007

**Published:** 2025-12-19

**Authors:** Viktor Kvasnytskyi, Anastasiia Zvorykina, Leonid Zvorykin, Constantine Zvorykin, Yevgenia Chvertko

**Affiliations:** Welding Department, National Technical University of Ukraine “Igor Sikorsky Kyiv Polytechnic Institute”, 37, Prospect Beresteiskyi, 03056 Kyiv, Ukraine; anastasiia.zvorykina@gmail.com (A.Z.); zvorykin1957@gmail.com (L.Z.); constantine.oleg@gmail.com (C.Z.); e.chvertko@kpi.ua (Y.C.)

**Keywords:** nitinol, thin wires, fusion welding, superelasticity, shape memory effect, intermetallides, oxides

## Abstract

The object of the study is a permanent joint of thin wires made of nitinol alloy. The problem of ensuring the formation of a joint of wires made of nitinol alloy was solved based on minimising changes in the structure of the welded joint material relative to the materials being joined. The properties of the welded joint material of the nitinol were studied using scanning electron microscopy and micro-X-ray spectral analysis. The studied permanent joint was obtained by TIG, microplasma (PAW) and capacitor discharge (CDW) welding. It was found that TIG welding can ensure the proximity of the microstructures of the wire and welded joint materials under conditions of sufficient protection in an argon atmosphere. Such TiNi welded joints have a welded joint material that retains its superelastic properties (within the limits of the shape memory effect). Capacitor discharge welding allows the joint to be brought closer to the required level of microstructure of the weld material. The results of mechanical tests demonstrated the limited capabilities of joints made of thin nitinol wires. At the same time, the appearance of only newly formed TiNi + TiNi_3_ eutectics in the weld material and a sufficient level of restoration of the welded joint shape give reason to consider capacitor discharge welding promising for joining thin nitinol wires. PAW leads to the formation of a significant amount of oxides in the weld and an increase in the number of Ti_2_Ni inclusions, which leads to brittle fracture of the welded joint even at low degrees of deformation. The results of the study can be used, in particular, for the manufacture of nitinol wire joints in medical devices.

## 1. Introduction

Alloy superelasticity is characteristic of materials that exhibit the shape memory effect—the ability to return to their original shape. This effect is associated with the austenite–martensite–austenite phase transition and is observed within a certain temperature range. The essence of the effect is that mechanical stresses during deformation of the material cause a thermoelastic austenite–martensite transition, while stress relief or temperature increase cause a reverse martensite–austenite transition and corresponding reverse deformations and shape restoration [1].

Nickel- and titanium-based alloys are typical representatives of shape memory alloys, which are also characterised by high corrosion resistance. This determines their use in transplant medicine [2].

In modern conditions, crystallography, thermodynamics of phase transformations and the mechanism of the shape memory effect in titanium-nickel alloys have long been sufficiently studied [3], and the main focus of research is on the practical use of such materials. Nitinol and other shape memory alloys are primarily used in medical equipment and devices, rocket and aircraft engineering, and space system instrumentation [4,5,6,7,8].

The requirements for such alloys are determined depending on the conditions of their use. These requirements usually include the temperature range of the effect, the number of cycles without significant residual deformation, and others.

For the successful creation of structures using shape memory effect materials, it is necessary to obtain permanent joints of elements made of these materials. Traditional methods for obtaining such joints are welding or soldering. Thus, research on joining superelastic nitinol wires by welding is promising for solving the urgent problem of finding ways to manufacture nitinol structures.

## 2. Literature Review and Problem Statement

The problems of welding shape memory alloys, in particular nitinol, have been addressed in many studies [9,10,11,12].

The results of [4] show that the thermal impact of laser radiation and recrystallisation of the material in the welding zone preserve the ability to restore the shape of the welded joint material. At the same time, the characteristic temperatures of direct and reverse martensitic transformation increase. The shift in the temperature intervals of martensitic transformation is associated with a change in the chemical or phase composition of the welded joint material as a result of laser radiation. This result is explained by a decrease in the concentration of nickel due to its evaporation or as a result of the formation of TiNi_2_, Ti_3_Ni_4_ and other inclusions in the welding zone. The observed changes in the temperature range of the shape memory effect in the welded joint material take the welding area beyond the material requirements, but outside the welding area, the functional properties of the material are preserved.

The influence of laser welding on the functional properties of TiNi was also investigated in [10]. Problems related to the reduction in the mechanical properties of the material in the welding zone were identified. To solve this problem, it is proposed to search for effective parameters of post-welding heat treatment. The authors of [10] did not find a final solution.

Research on plasma welding of nitinol with nitinol [11] revealed that cracks occur in the weld area, with a more brittle Ti_2_Ni phase. No changes in the ratio of nickel and titanium concentrations in the weld material were found. It was noted that the strength of the welded material is significantly lower than that of the welded material.

A promising direction for research into nitinol welding is solid-state welding [12,13,14]. The use of such joining methods avoids many problems associated with changes in elemental composition due to the evaporation of elements, the formation of new phases, and the emergence of metastable structural states. At the same time, diffusion welding is a lengthy process and occurs at high austenite temperatures, friction welding causes structural destruction as a result of mechanical mixing of the material in the joint area, and explosion welding leads to deformation and significant changes in the structure of the material. All these types of welding can only be used in limited types of structural elements.

Work [15] describes TIG welding of nitinol wire and sheet metal. It shows that after welding, the manufacturing cycle of the welded joint must include heat treatment to bring the properties of the welded joint and the base material closer together. Most studies conducted later confirm the validity of this approach, but the identity of the properties of the welded joint material and the base material for nitinol has not yet been achieved for any welding method.

Fundamental solutions for joining shape memory metal elements must preserve the ability of unloaded materials under external stress to accumulate up to 10–15% of deformation energy, which, in the case of superelasticity, returns to its previous shape when the external load is removed [9].

Thus, the main problems that exist when welding nitinol are changes in the elemental and phase composition and microstructure in the weld area and in the heat-affected zone. This leads to changes in the parameters of phase transitions that ensure the realisation of the shape memory effect (in particular, superelasticity) and, accordingly, to different degrees of realisation of this effect in materials in the weld area and the base material. This negative effect on the properties of the welded joint is often compounded by the formation of brittle intermetallics, near which the existing deformation stresses can cause the existence of thermoelastic martensite, which limits the completeness of shape recovery. The process of formation of Ti_2_Ni, TiNi_3_ intermetallides in nitinol after welding is practically irreversible. This leads to a local change in the ratio of titanium and nickel and a corresponding change in the shape memory effect. All these features make it impossible to determine a single universal type of welding that can be recommended for nitinol. In addition, the specific requirements for a product manufactured by welding in most cases impose their own restrictions on the choice of welding method and the corresponding post-welding heat-treatment cycle.

All this allows us to conclude that research devoted to determining the dependence of the welded joint material structure on welding conditions is relevant. This is the way to determine the characteristics of the welding thermal cycle, which will ensure the formation of a welded joint with an optimised joint structure that meets operational requirements.

## 3. The Aim and Objectives of the Study

The aim of the study is to ensure the formation of a thin nitinol wire joint by creating conditions for the formation of a welded joint material structure that is as close as possible to the base material. This will make it possible to recommend a welding mode for obtaining a high-quality welded joint with a structurally homogeneous weld that retains the properties of the material with a shape memory effect without thermal stimulation.

To achieve the aim of the work, the task to determine the effect of TIG, PAW and CDW methods on the structure and ability to restore the shape of a welded joint of thin nitinol wire without additional thermal impact was set.

## 4. The Study Materials and Methods

### 4.1. Characteristics of the Materials Being Joined and Methods of Obtaining Permanent Joints

**Figure 1:**
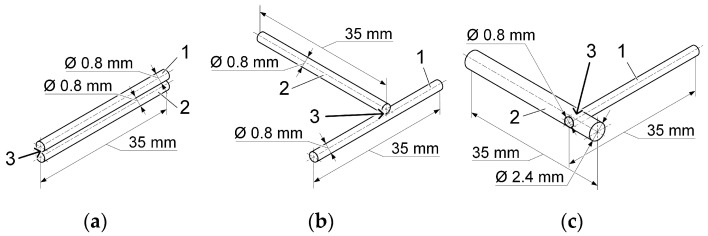
Scheme of assembling samples for welding: (**a**)—TIG and PAW methods; (**b**)—CDW butt joints; (**c**)—CDW joint with diametrically opposed holes with a diameter of 0.8 mm in a 2.4 mm diameter sample; 1, 2—samples before welding; 3—welded joint area.

The experimental part of the research consisted of making permanent joints between thin wires made of Nitinol alloy. Nitinol alloy wires with diameters of 0.8 mm and 2.4 mm were used to make the joints. The joints were made by arc welding with a non-consumable electrode in an inert gas environment, microplasma welding, and capacitor discharge welding. Lap joints were obtained by melting the end surfaces of wires arranged in parallel using an inert gas non-consumable electrode and microplasma welding. Figure 1a shows the diagram of the wire assembly for arc welding with a non-consumable electrode in an inert gas and for microplasma welding. Lap joints (Figure 1b) and joints of a larger-diameter wire with a diametrically located hole into which a smaller-diameter wire was inserted (Figure 1c) were obtained using capacitor discharge welding.

Capacitor discharge welding is characterised by minimal thermal impact, as it is a pressure welding method. In capacitor discharge welding, the energy stored in a capacitor bank is used to heat and join metal parts. The stored energy is released locally, with a discharge time of 0.001 to 0.005 ms, which ensures a high density of welding current at the contact point of the parts being joined. The process ensures high welding accuracy, low power consumption, minimal deformation of parts, and the ability to weld small and thin-walled workpieces. The chemical composition of nitinol alloy wires is shown in Table 1.

Arc welding with a non-consumable electrode in an inert gas environment (TIG) and microplasma welding (PAW) were performed using high-purity argon (Ar > 99.998% vol.) produced by Linde as a shielding and plasma-forming gas.

### 4.2. Preparation of Samples for Research

To obtain permanent joints of thin nitinol alloy wires, samples with diameters of 0.8 mm and 2.4 mm 35 mm long manufactured by mechanical processing were used. In wire samples made of nitinol alloy with a diameter of 2.4 mm, through holes with a diameter of 0.8 mm were drilled in the diametrical plane, into which a wire with a diameter of 0.8 mm was inserted (Figure 1c).

After machining, the samples were washed with acetone and treated with ethyl alcohol. Immediately before joining the sections of the samples to be welded, they were mechanically treated using P1000 sandpaper. After fine grinding, the samples were washed with ethyl alcohol and placed in a device for fixing the samples during welding.

### 4.3. Welding with a Non-Consumable Electrode in an Inert Gas Environment and Microplasma Welding

Permanent joints of thin nitinol alloy wires were made according to the scheme shown in Figure 1a, using an electric arc as a heat source by welding with a non-consumable tungsten electrode in an inert gas environment and microplasma welding using argon as a plasma-forming and shielding gas. Welding was performed without the use of filler wires.

Welding with a non-consumable tungsten electrode in an inert gas environment, as well as microplasma welding, was performed using indirect heating, when the welded samples were not connected to the electrical circuit. During welding with a non-consumable tungsten electrode in an inert gas environment, the electric arc burned between the central tungsten electrode of the welding torch and an additional tungsten electrode. The distance between the electrodes (the length of the electric arc) was between 4 mm and 6 mm. This approach was chosen in order to reduce the amount of heat input and overheating of the weld pool formed from the base metal.

Another feature of the TIG welding process was the use of a 19.5 mm diameter Jambo gas lens to provide high-quality protection from the effects of the surrounding environment—air. The use of a gas lens increases the volume of space protected by inert gas, reduces the flow rate of the protective inert gas and the degree of turbulence in its flow, which helps to reduce the “suction” of air and its mixing with argon.

A fully digital, digitally controlled inverter-type welding power source designed for TIG welding, MagicWave 2200 Job (Fronius International GmbH, Wels, Austria), was used as the power source. The MagicWave 2200 Job power source allows you to adjust the welding current for TIG welding in the range from 3 A to 220 A. The no-load voltage is 88 V. The power source is equipped with a built-in pulse contactless electric arc ignition unit with an ignition voltage of 9.5 kV, which ensures easy and reliable contactless ignition of the electric arc and makes it possible to use it for microplasma welding.

A standard WIG welding torch (Fronius International GmbH) was used for TIG welding. The diameter of the tungsten electrode is 2.6 mm.

**Figure 2:**
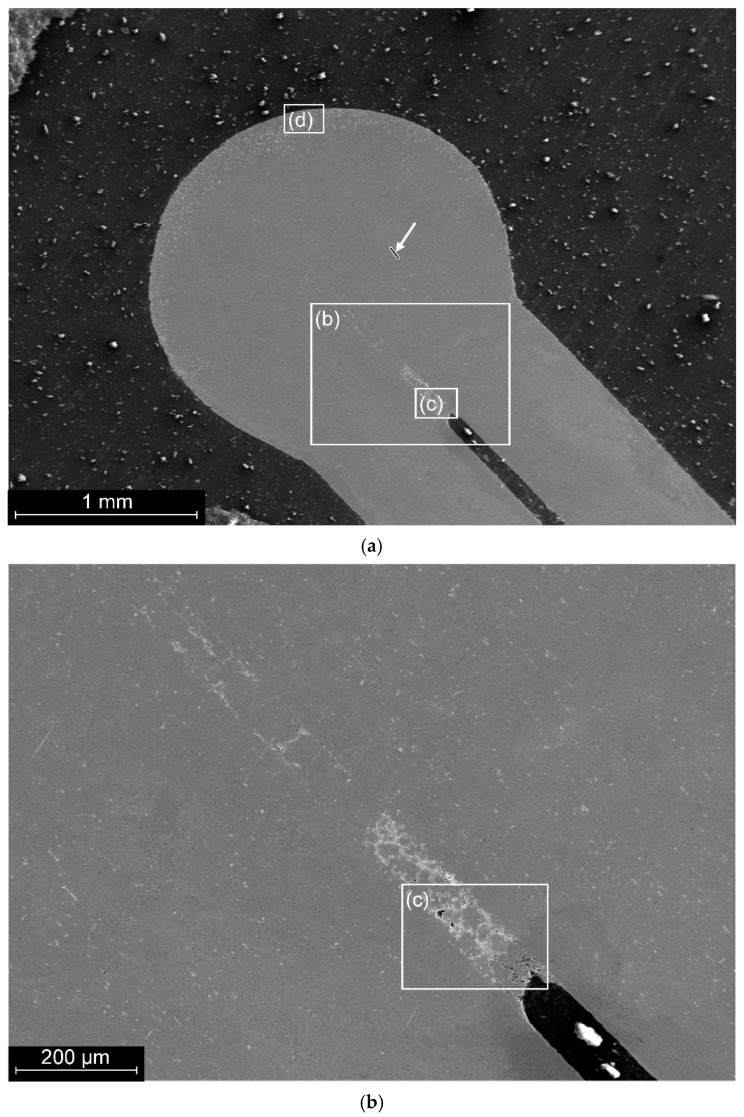

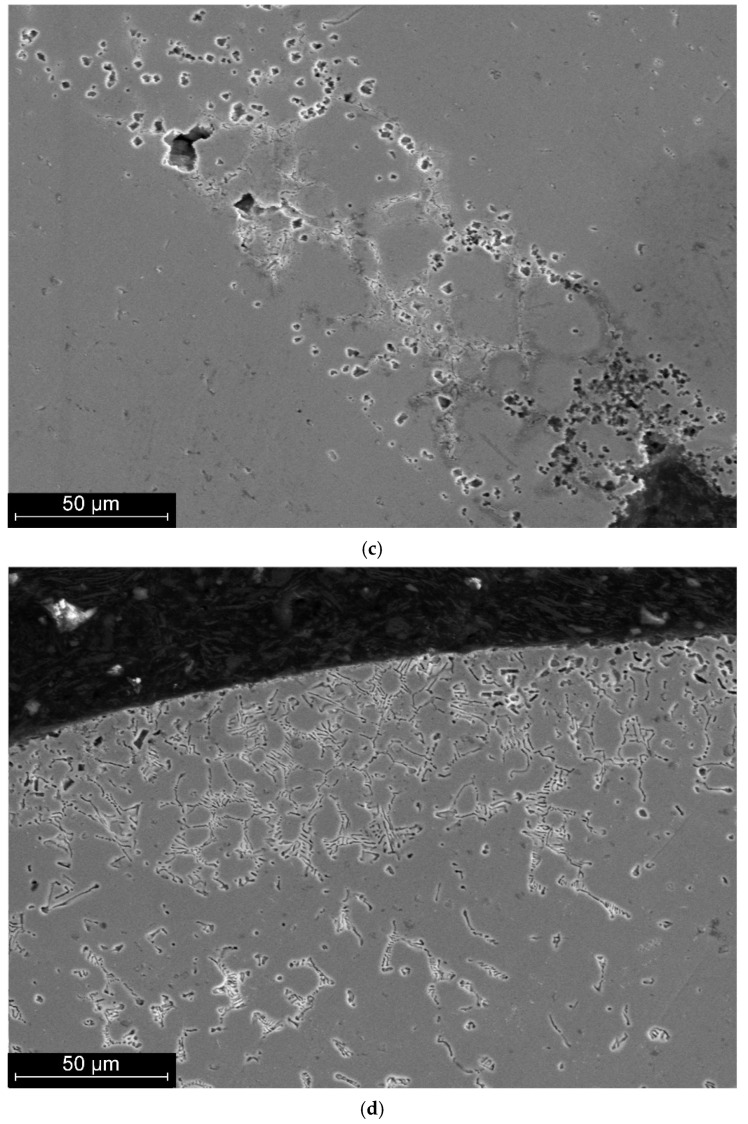
The longitudinal section of a welded joint of a sample made by TIG welding: (**a**)—general view; arrow points the line of EDS-scanning, see Figure 3a; (**b**)—structure of the root zone; (**c**)—microstructure of the material in the root zone; (**d**)—microstructure of the surface zone.

A direct-action plasmatron, developed by NVC “PLAZER” LLC, Kyiv, Ukraine (Figure 2), was used as a heat source for microplasma welding.

A distinctive feature of the microplasma welding process was the use of a compressed microplasma arc of indirect action to heat the product. The plasma electric arc burned between the central tungsten electrode of the plasma torch and an additional tungsten electrode included in the welding electric circuit. The distance between the electrodes (the length of the compressed plasma electric arc) was between 5 mm and 6 mm. The welded samples were placed in the arc gap during heating. Depending on the parameters of the welding current and the time spent in the plasma arc heating zone, the heating rate and the volume of the weld pool were changed.

The recommended values for the parameters of non-consumable electrode welding in an inert gas environment and microplasma welding are given in Table 2.

**Figure 3:**
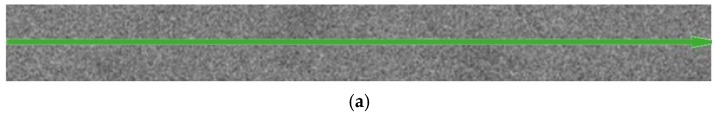

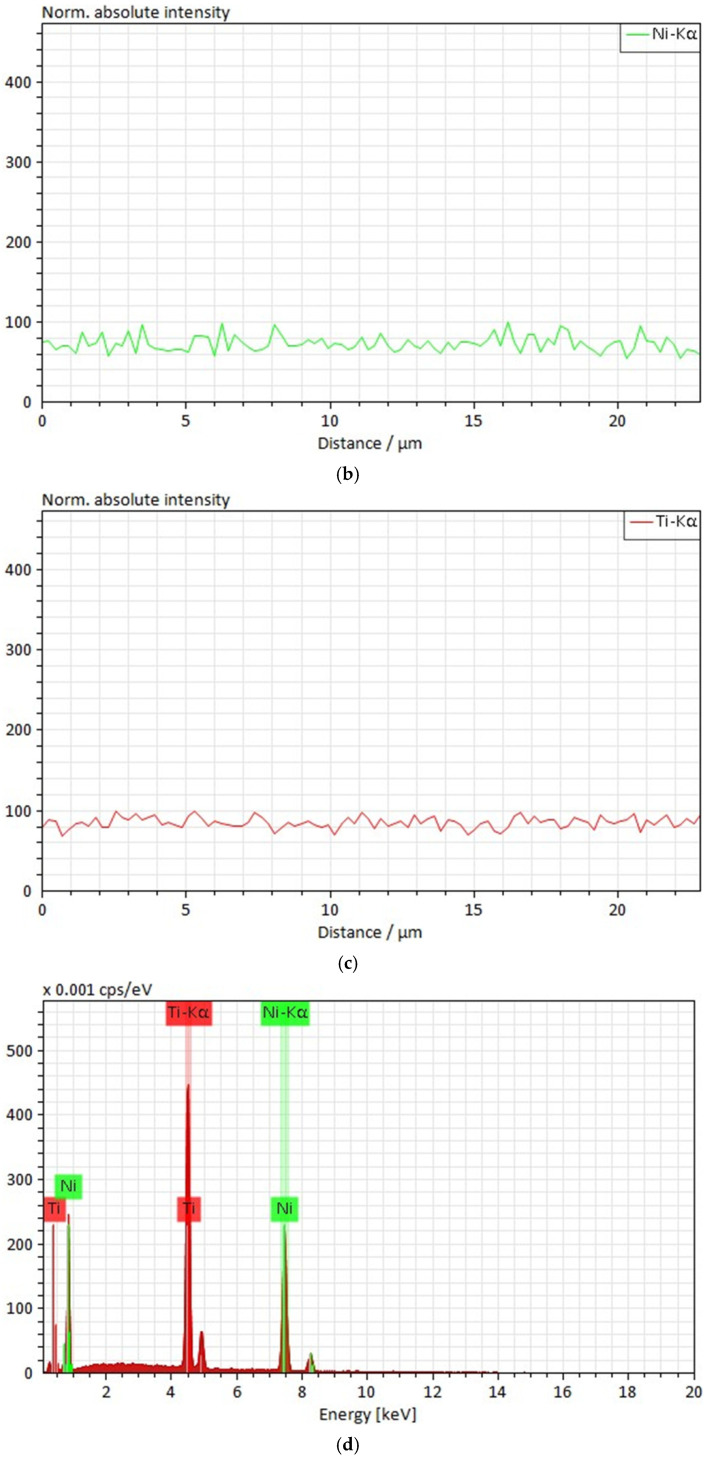
Results of micro-X-ray scanning of a welded joint of a nitinol sample made by TIG welding: (**a**)—scanning line; (**b**)—intensity of characteristic Ti radiation along the scanning line; (**c**)—intensity of characteristic Ni radiation along the scanning line; (**d**)—micro-X-ray spectrum along the scanning line.

Figure 3 shows the results of micro-X-ray scanning of a welded joint of a nitinol sample made by TIG welding.

### 4.4. Capacitor Discharge Welding

Like any method of resistance welding with accumulated energy, capacitor discharge welding is a technological process in which a permanent joint between metal parts is formed due to the release of heat in them during the passage of welding current, which occurs when using a pre-accumulated amount of electrical energy (in this method, a charged capacitor bank). In capacitor discharge welding machines, energy is stored in the capacitor bank during charging from a direct current source (generator or rectifier) and then used for welding.

Capacitor discharge welding of thin nitinol alloy wires was performed on a TKM-15U4 spot capacitor discharge welding machine. This type of machine belongs to the category of transformer-type spot capacitor discharge welding machines, where the capacitors discharge into the primary winding of the welding transformer, in the secondary circuit of which the pre-compressed parts to be welded are located. Thus, with the help of the TKM-15U4 machine, it is possible to perform resistance spot welding with the option of adjustable preheating of the metal in the joint area. The technical characteristics of the TKM-15U4 spot capacitor discharge welding machine are given in Table 3.

To obtain welded joints of various designs, welding electrodes of various geometric shapes and sizes were manufactured from bronze by mechanical processing. The need to manufacture such electrodes is related to the requirements for ensuring a reliable supply of welding electric current to the welded samples and applying a compressive force in a direction perpendicular to the weld spot formation area, which is formed at the contact boundary of the sample surfaces.

Resistance spot capacitor discharge welding of wire elements with a diameter of 0.8 mm between each other and with 2.4 mm made of nitinol alloy was carried out according to the modes given in Table 4. Overlapping joints were formed (see Figure 1b). The angle between the connected wires was 90 degrees. Another joint was made between a 2.4 mm-diameter Nitinol alloy wire and a 0.8 mm diameter through hole drilled in the diametrical plane. Then, a 0.8 mm diameter wire was inserted into this hole (Figure 1c). The welding current was applied separately to the 2.4 mm diameter sample and the 0.8 mm diameter sample. During welding, a compressive force perpendicular to the plane formed by the axes of the joined samples was applied.

The transformation coefficient of the welding transformer K_tr_ was 100 in all cases.

### 4.5. Investigation of the Microstructure and Mechanical Properties of the Material in Welded Joints

To perform metallographic and micro-X-ray spectral analysis of welded nitinol wire samples, they were pressed into bakelite in a steel cylindrical mandrel. The samples were ground mechanically until their diametrical cross-section was achieved. Subsequently, the samples were mechanically polished to a roughness of R_z_~0.2 µm.

Chemical etching of the samples to reveal their microstructure was carried out in a 49% nitric acid (HNO_3_) solution with the addition of 18% HF and 23% water, followed by rinsing with ethyl alcohol.

The samples were examined using a TESCAN VEGA 3 scanning electron microscope (Brno, Czech Republic) with an energy-dispersive X-ray analyser.

The research was conducted in secondary (SE) and backscattered electron (BE) modes. Secondary electrons are generated in thin surface layers (1–5 nm), so this type of radiation is effective for obtaining information about surface relief. Back-scattered electrons are reflected from the sample surface due to elastic scattering (within a layer of 1–5 µm), with the signal intensity depending on the average atomic number of the elements in the studied area. This made it possible to study the nature of changes in the shape and size of individual fragments and elements of the surface structure.

The composition of the elements in the test sample was determined by energy dispersive analysis (EDS), which provides microanalysis of dispersed particles and phases. Microanalysis provides a high degree of locality due to its use in conjunction with an electron microscope. The minimum diameter of the analysed area is at least 1 µm.

One of the main advantages of an energy dispersive spectrometer is the high speed of spectrum accumulation, the ability to perform short-term quantitative elemental analysis, and the rapid acquisition of element distribution maps across the sample surface.

The method applied allows to define the content of most elements in the range of 0.1–0.5 mass %. For local analysis of elements with low concentrations, a second spectrometer with wavelength dispersion is used.

To determine the mechanical properties of the material in welded joints, the welded joint area was mechanically fixed and one of the wires was deflected at an angle of 30° and then returned to its original state. The deflection and return were repeated 10 times until destruction or residual deformation of more than 4° occurred.

Samples that successfully passed the first cycle of mechanical tests were studied under conditions of deflection and return 10 times at an angle of 60° until destruction or the occurrence of residual deformation greater than 4°.

## 5. Research Results

### 5.1. Structural and Mechanical Characteristics of Joints Made by TIG Welding

A general view of the structure of a typical type 1 nitinol joint made by TIG welding is shown in Figure 2a.

The diameter of the weld was 2.2 mm.

A preliminary analysis of the surface did not reveal any macrodefects in the material, but showed that the area on the side of the weld that is furthest from the joined wires has a structure that differs from that of the base material and the weld. Deviations from the general structure are also observed in the root zone of the weld (Figure 2b).

A more thorough analysis of the root zone material revealed the presence of phase formations ranging in size from 1 to 20 microns (Figure 2c).

In the area of the outer surface of the weld, phase formations with grains of 10–20 µm were observed only in the near-surface zone at a depth of up to 15 µm. At the same time, the near-surface layer of material with a thickness of up to 200 µm is characterised by the presence of eutectic-type structural formations (Figure 2d).

Analysis of eutectic formations in the surface zone showed the presence of up to 6 wt.% of oxygen in them (Table 5), which made it possible to identify the NiTi_2_O phase in this zone and assume the existence of Ti_2_O.

Scanning with an electronic probe along the wire axis to in the weld zone (Figure 2a and Figure 3) showed a homogeneous distribution of titanium and nickel close to the equiatomic composition.

**Figure 4:**
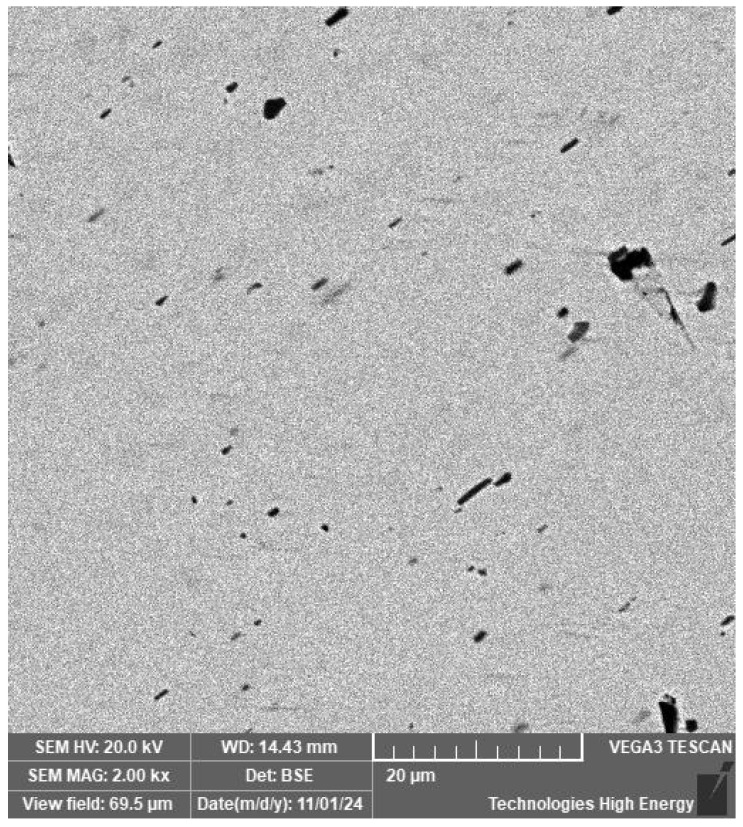
Point inclusions in the structure of nitinol.

The structure of the wire material, welded joint and heat-affected zone is characterised by the same metallographic structure with point inclusions ranging in size from 1 to 5 µm (Figure 4).

**Figure 5:**
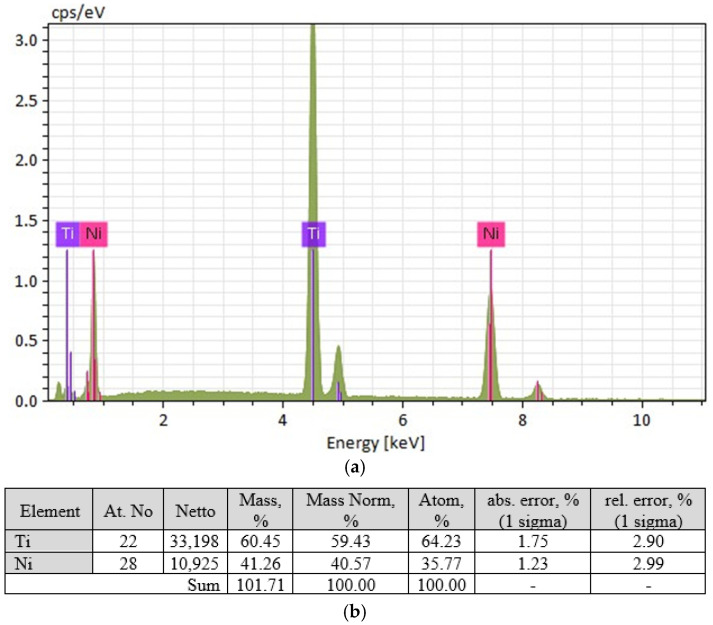
Results of micro-X-ray analysis of large inclusions in the welded joint of a nitinol sample made by TIG welding: (**a**)—micro-X-ray spectrum for large inclusions; (**b**)—elemental composition characteristics for large inclusions.

The ratio of nickel and titanium concentrations in larger inclusions, as revealed by micro-X-ray analysis (Figure 5), allows the identification of the Ti_2_Ni phase.

Mechanical tests of welded joints were performed by bending welded wires at angles of 30° and 60°. According to the test results, all welded joints withstood bending cycles at an angle of 30° and failed before reaching an angle of 60°.

**Figure 6:**
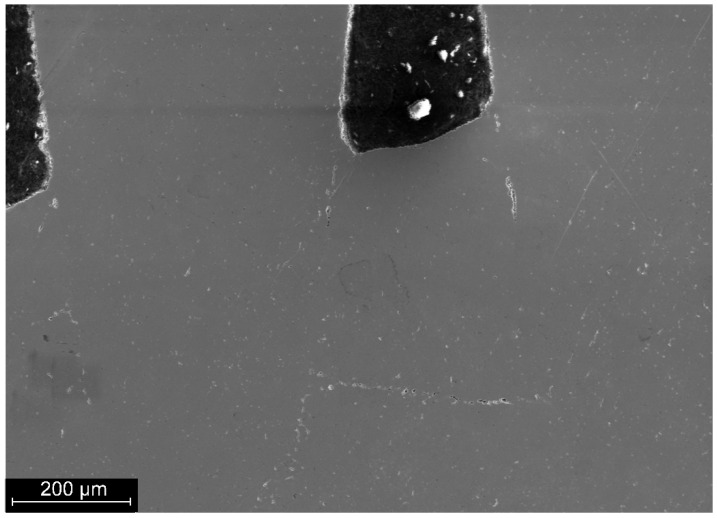
Microstructure of the root zone of a welded joint of a nitinol sample made by TIG welding under increased atmospheric insulation.

Based on the hypothesis of insufficient atmospheric insulation during the jointing process, the argon supply was subsequently increased to 13 L·min^−1^. Analysis of the root zone structure of the welded joint showed that increasing the atmospheric protection of the joint during welding prevented the formation of a critical region with oxygen-containing phases in the root zone of the weld (Figure 6).

In the material of the formed weld, the ratio of nickel and titanium concentrations remains constant within the range of 51.02–52.94 at.% Ti, 47.06–48.98 at.% Ni, which is within the measurement error range and corresponds to the passport characteristics (Table 1). The number and distribution of Ti_2_Ni inclusions in the TiNi base in the welded joint material and the heat-affected zone does not differ from the welded wire material.

The strength tests carried out on the welded joint showed that the welded wires could be bent repeatedly at angles of both 30° and 60° without breaking and residual deviation at an angle of no more than 4°.

### 5.2. Structural and Mechanical Characteristics of Joints Made by Microplasma Welding

**Figure 7:**
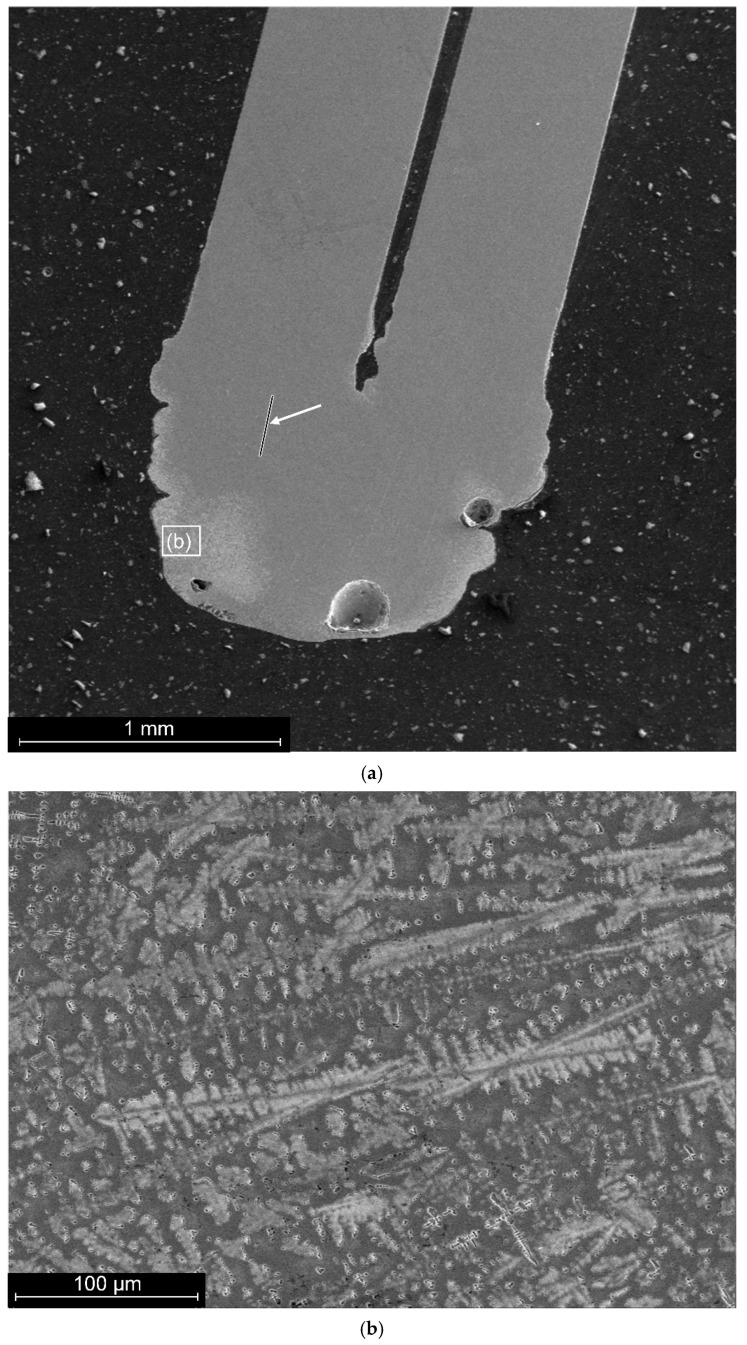
Longitudinal cross-section of a welded joint of a nitinol sample made by PAW: (**a**)—general view; arrow points the line of EDS-scanning; (**b**)—microstructure of the surface zone.

Nitinol wire with a diameter of 0.8 mm was welded in pairs. A general view of the cross-section of a typical joint is shown in Figure 7a.

**Figure 8:**
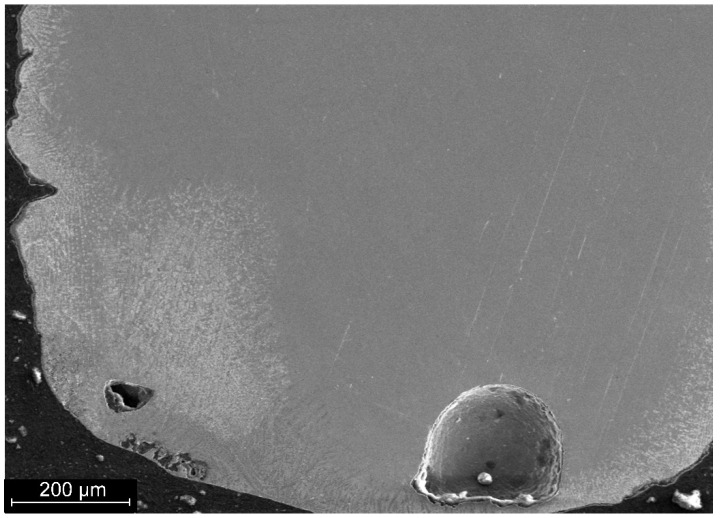
Structure of the surface zone of a welded joint of a nitinol sample made by PAW.

Preliminary analysis of the surface shows the presence of macrodefects in the material, in particular macropores with a size of 30–100 µm. The surface area of the weld has a structure that differs from the structure of the base material and the weld (Figure 8). The thickness of the layer with the altered structure reaches 400 µm. Deviations from the overall structure are also observed in the root zone of the weld.

The microstructure of the layer of material with structural changes up to 400 µm thick is characterised by a dendritic structure (Figure 7b).

Analysis of phase formations in the surface zone, which is the basis of dendritic grains, showed the presence of up to 20 at.% oxygen in them. The oxide phases of nickel and titanium have a crystallisation temperature significantly higher than 1310 °C, at which TiNi crystallisation begins.

**Figure 9:**
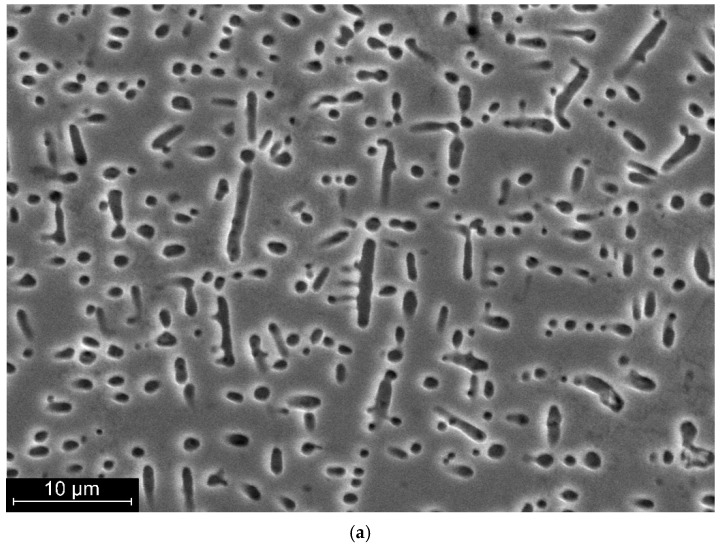

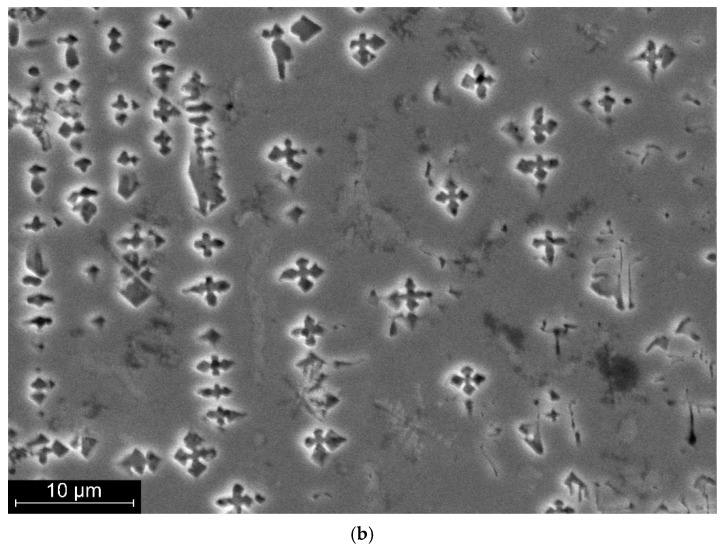
Characteristic appearance of oxide phase formations in the near-surface zone of a welded joint of a nitinol sample made by PAW: (**a**)—at depths up to 300 µm; (**b**)—at depths from 300 µm.

The morphology of oxide phases varies with depth from the surface. At shallow depths, phase formations are characterised by a punctate or lamellar appearance (Figure 9a). At greater depths, such phases have a cross-like appearance (Figure 9b).

The elemental composition of the oxide phase formations corresponds to the composition of the NiTi_2_O phases detected after TIG welding with insufficient argon protection. At the same time, the density and shape of the phase formations differ significantly. The nature of the oxide phase formations indicates a sufficiently high concentration of oxygen in the near-surface region of the weld and a high rate of its crystallisation.

**Figure 10:**
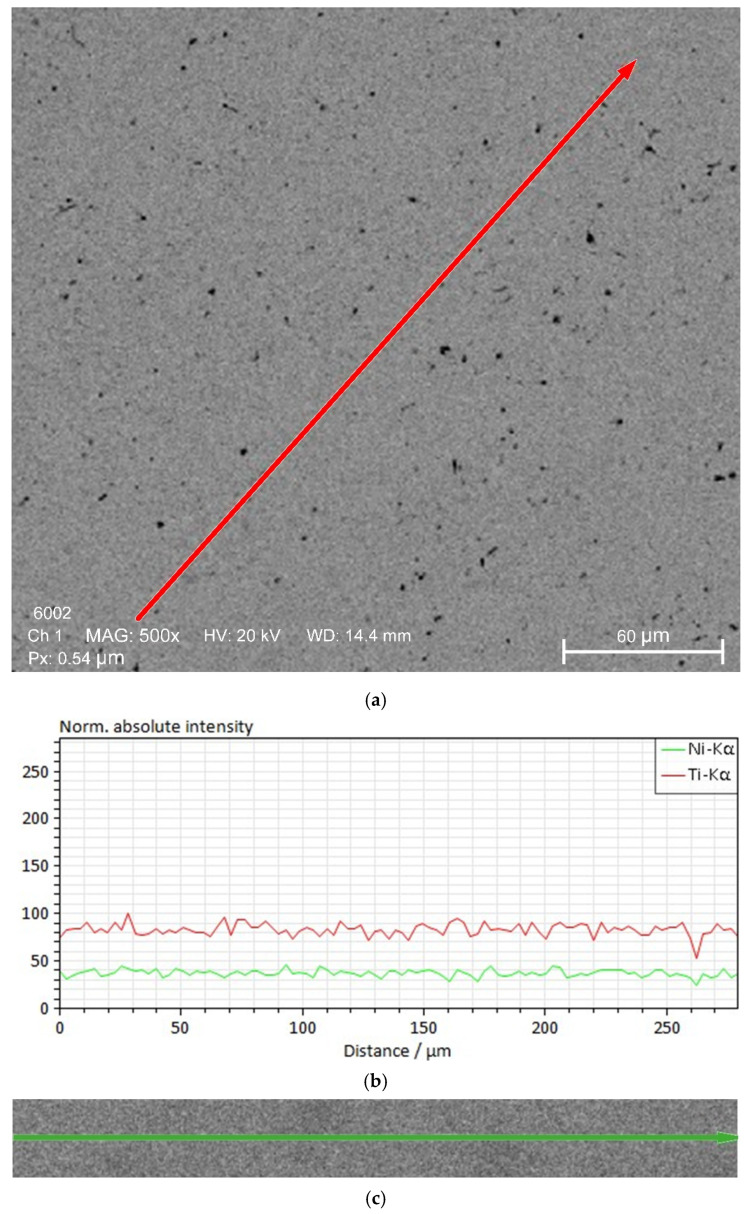
Results of micro-X-ray scanning of a welded joint of a nitinol sample made by PAW: (**a**)—scanning line in Ti radiation; (**b**)—change in the intensity of Ti and Ni radiation; (**c**)—scanning line in Ni radiation.

Scanning with an electron probe along the wire axis in the weld zone (Figure 7a and Figure 10) indicates a homogeneous distribution of titanium and nickel close to the composition of 45 wt.% Ti, 55 wt.% Ni.

**Figure 11:**
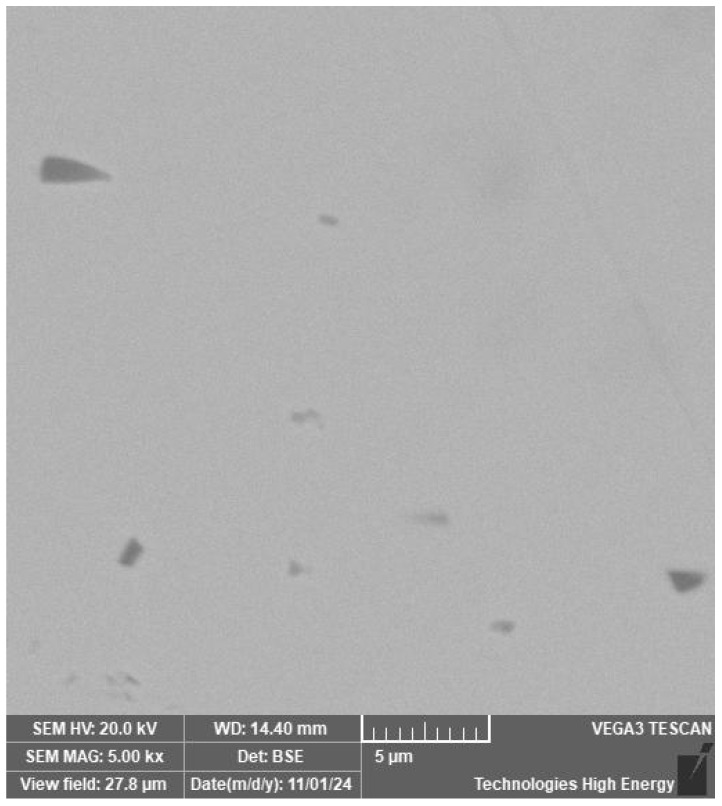
Point inclusions in the structure of nitinol wire.

Dark-coloured phase inclusions are observed in the nitinol material (Figure 11). The density of such inclusions in the nitinol wire area is 1.5 times lower than in the weld area. The average size of such inclusions in the wire material is from 1 to 3 µm. The structure of the welded seam material and the heat-affected zone is characterised by a metallographic structure with point inclusions larger than 1 to 5 µm.

**Figure 12:**
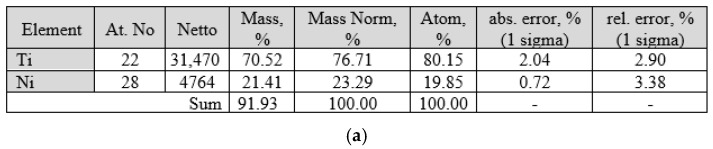

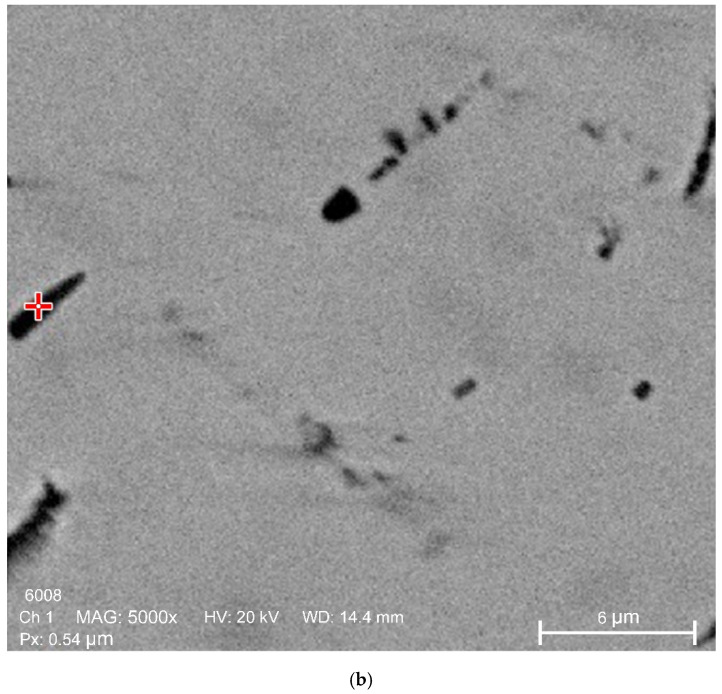
Results of micro-X-ray analysis of large inclusions in the welded joint of a nitinol sample made by PAW: (**a**)—elemental composition characteristics for large inclusions; (**b**)—view of the inclusions in the welded joint that were analysed.

The ratio of nickel and titanium concentrations in larger inclusions, as revealed by micro-X-ray analysis (Figure 12), allows the identification of the Ti_2_Ni phase.

Mechanical tests of welded joints were performed by bending welded wires at angles of 30° and 60°. According to the test results, 30% of welded wires were destroyed during repeated bending of welded wires, and all welded joints were destroyed before reaching the specified angle of 60°.

The strength tests of welded joints obtained under conditions of increased atmospheric insulation (under conditions of increased argon flow rate) did not lead to satisfactory mechanical characteristics.

### 5.3. Structural and Mechanical Characteristics of Joints Made by Capacitor Discharge Welding

**Figure 13:**
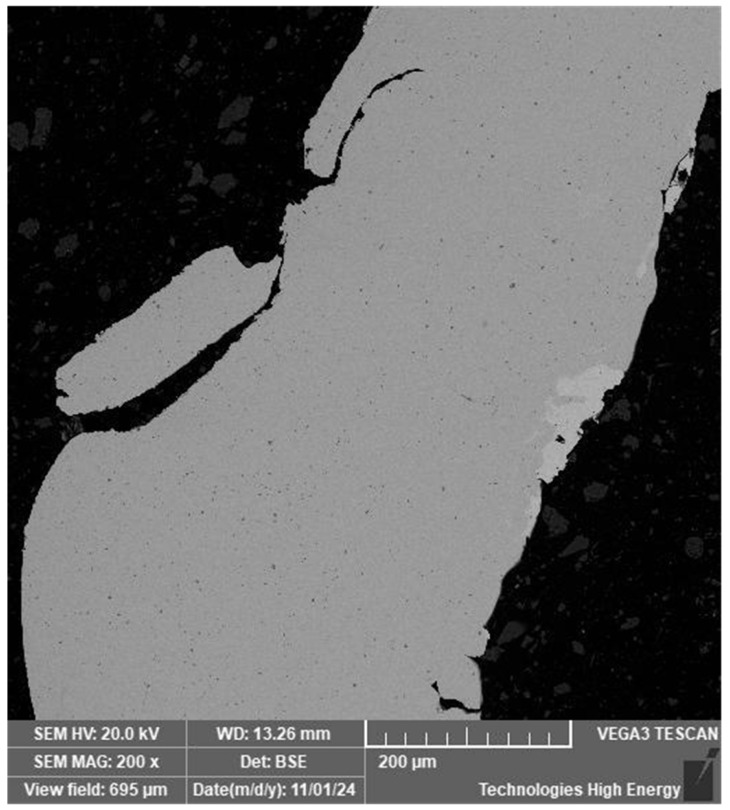
View of the structure of the longitudinal section of the welded joint of the nitinol sample made by CDW.

The general view of the structure of the welded joint of a typical joint obtained by CDW of superimposed wires with a diameter of 0.8 mm is shown in Figure 13.

The thickness of the welded wires as a result of deformation during welding is close to 0.55 mm.

**Figure 14:**
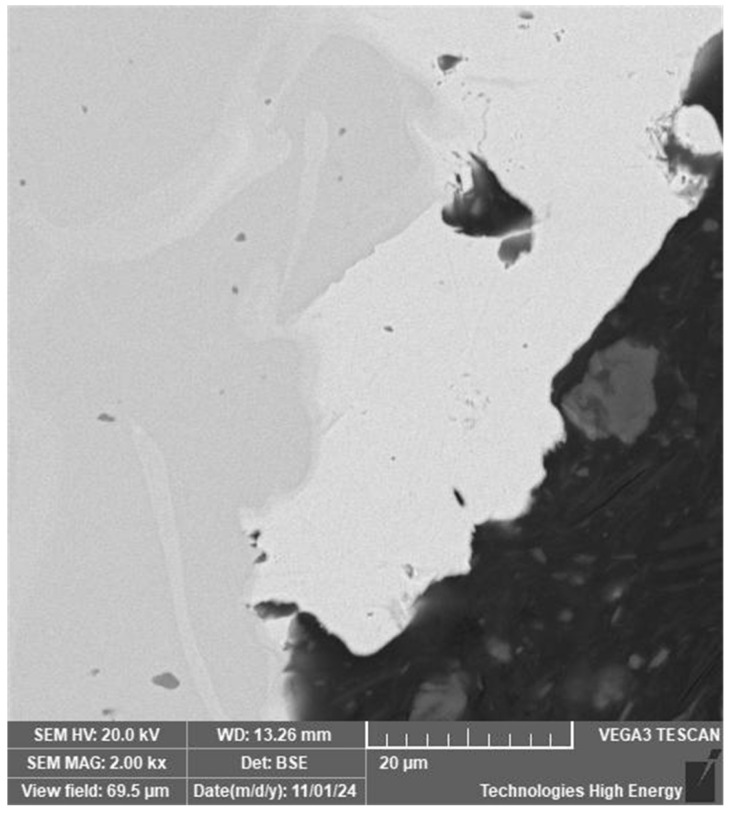
Structure of the surface zone of a welded joint of a nitinol sample made by CDW.

A preliminary analysis of the surface revealed the presence of macrodefects in the material, in particular macro-cracks measuring 150 µm and 250 µm. On the one hand, the surface area of the weld has a structure that differs from the structure of the base material and the weld (Figure 14). The thickness of this layer reaches 60 µm.

**Figure 15:**
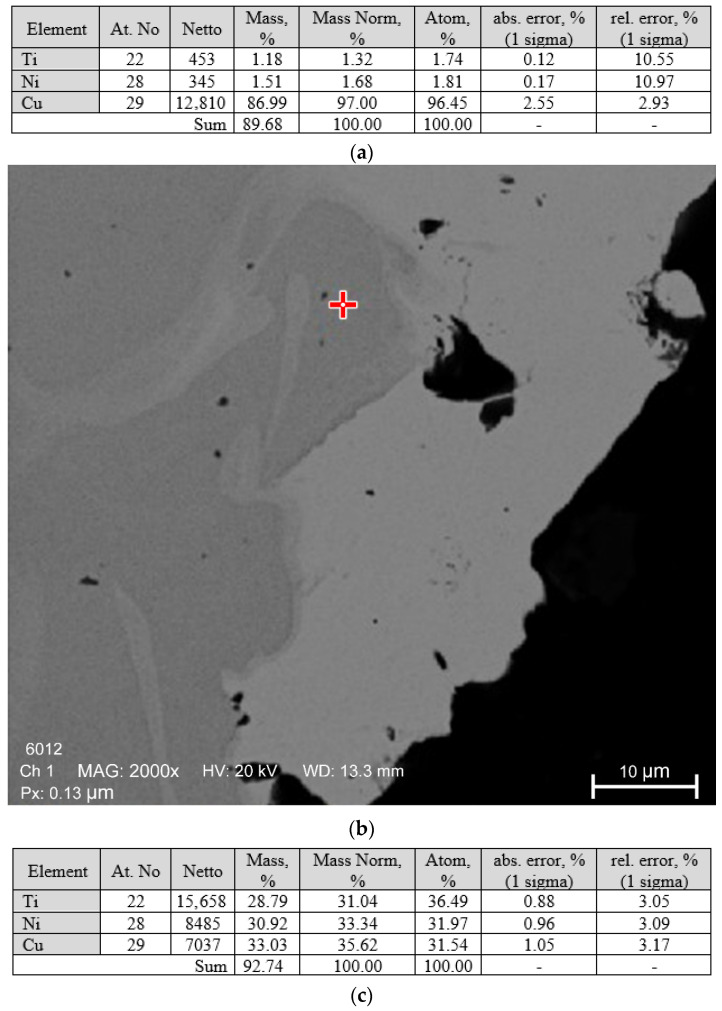
Results of micro-X-ray spectral analysis of the surface zone of the welded joint of the nitinol sample made by CDW: (**a**)—characteristics of the elemental composition of the surface material; (**b**)—surface area analysed; (**c**)—characteristics of the elemental composition of the surface area.

Micro-X-ray spectral analysis of different areas of the surface layer of the welded joint material showed the presence of copper (Figure 15). This indicates the transfer of copper electrode material to the welded joint.

The welded joints obtained have unsatisfactory mechanical properties, since repeated bending of the welded wires at an angle of 30° resulted in destruction. It should be noted that the presence of copper imposes additional restrictions on the use of the joint, in particular for medical devices.

**Figure 16:**
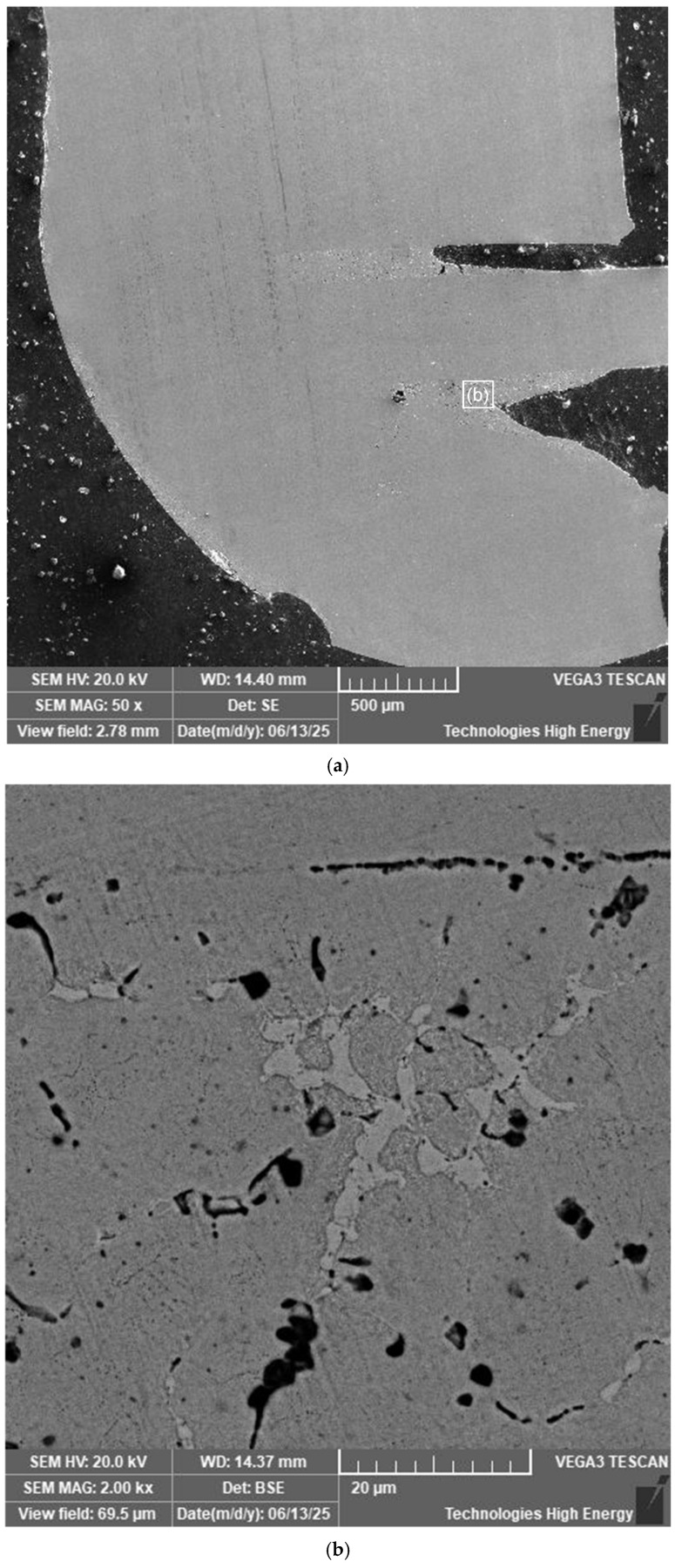
The longitudinal cross-section of a welded joint made by capacitor discharge welding of nitinol wires in a through hole: (**a**)—view of the structure; (**b**)—microstructure of the welded joint material in the area of radial contact zone of a 0.8 mm diameter wire in a through hole in a 2.4 mm diameter wire.

Figure 16a shows a welded joint made at the junction of a wire with a diameter of 0.8 mm with a wire diameter of 2.4 mm with a hole diameter of 0.8 mm preformed in the diametrical plane of the sample (perpendicular to the axis of the sample with a diameter of 2.4 mm).

In the end area of the 0.8 mm diameter wire in the hole in the 2.4 mm diameter wire, the welded seam does not differ in structure from the wire material. Structural changes are observed in the welded seam material in the area of radial contact between the 0.8 mm diameter wire and the hole surface. No defects such as pores or cracks were found in the weld zone, but the presence of light and dark phase inclusions was established (Figure 16b).

**Figure 17:**
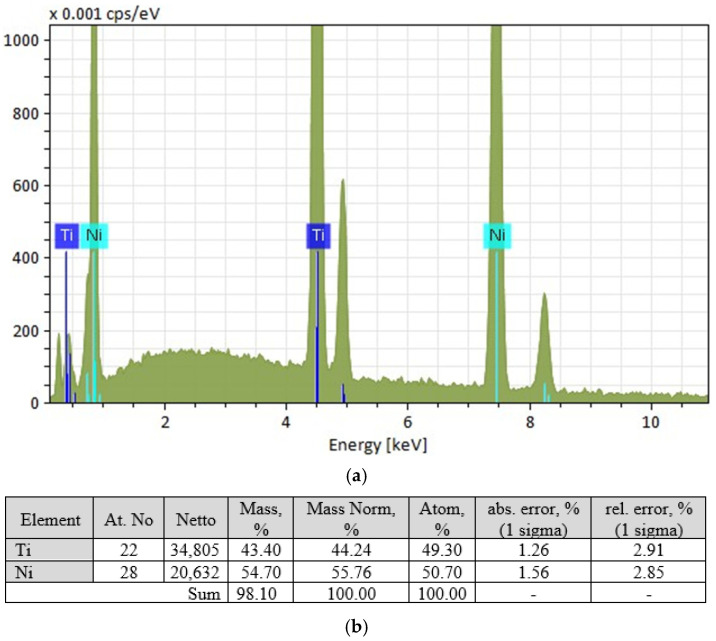
Results of micro-X-ray spectral analysis of the grey phase: (**a**)—spectral characteristics of the phase; (**b**)—characteristics of the elemental composition of the phase.

Micro-X-ray spectral analysis showed that the base material (grey phase) has a composition close to equiatomic Figure 17.

**Figure 18:**
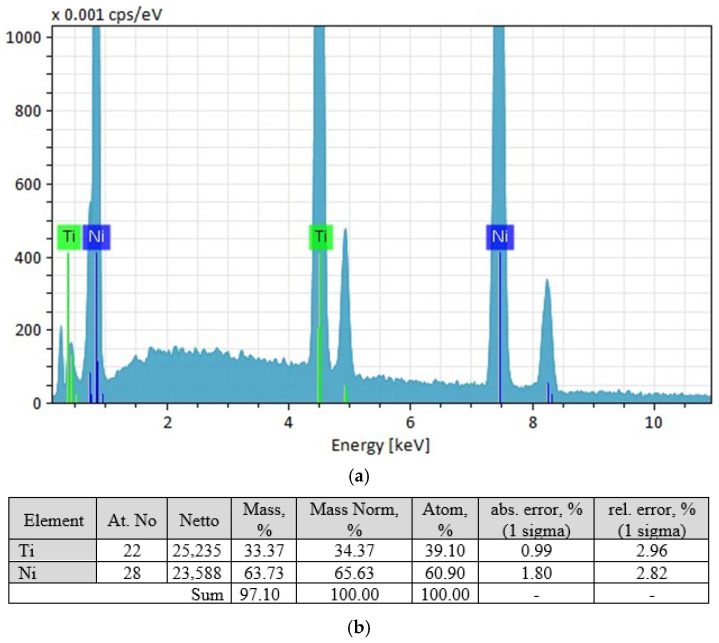
Results of micro-X-ray spectral analysis of the light-coloured phase: (**a**)—spectral characteristics of the phase; (**b**)—characteristics of the elemental composition of the phase.

The light-coloured phase has a composition close to TiNi_2_ (Figure 18), but this phase does not correspond to the phase diagram. The equilibrium phase is TiNi_3_, which allows the light-coloured grains to be identified as a eutectic of TiNi + TiNi_3_.

The elemental composition of the dark phase is within the range of 46.83 at.% Ti 53.07 at.% Ni—54.11 at.% Ti 44.89 at.% Ni.

Analysis of the mechanical properties of the obtained compounds showed that under conditions of repeated bending of samples welded wires at an angle of 30°, shape recovery is observed. When bent at an angle of 60°, 40% of the samples were destroyed, and another 10% had shape recovery of up to 70%.

## 6. Discussion of the Results

To achieve the goal of forming a thin nitinol wire joint, it is advisable to obtain a welded joint without defects and negative phase components of the weld material and heat-affected zone. Such components include intermetallics and other phase formations that are equilibrium and, accordingly, cannot be eliminated by further heat treatment without melting. A certain number of phases of this type are found in welded materials, but they are not detected by X-ray phase analysis and are recorded by metallographic analysis as point inclusions of low concentration and size.

The results of studying the structural characteristics of TIG-welded joints showed that the critical factor affecting strength and superelasticity is the formation of oxide phases (Figure 2c) in the root area of the weld. In the presence of such phases, the joint brittle fails under mechanical stress. Increasing the degree of protection of the weld material from atmospheric oxygen with inert argon allows to eliminate the formation of a significant amount of phase inclusions (Figure 8) containing oxygen (NiTi_2_O, Ti_2_O). It should be noted that the presence of oxide phases in the outer surface layer cannot be considered a negative factor affecting the quality of the welded joint, since they are not located in the critically loaded area during mechanical testing. At the same time, the presence of such phases on the surface increases the biological compatibility of the product [16].

Comparative metallographic analysis of welded joint materials (Figure 6) and nitinol wire (Figure 12) gives no reason to believe that TIG welding leads to an increase in the number or size of Ti_2_Ni intermetallic compounds. Positive results of mechanical tests of the welded joint and shape recovery with acceptable residual deformation give reason to believe that TIG welding under the recommended conditions with improved argon protection (use of a gas lens and increased protective gas consumption to 13 L·min^−1^ protective gas consumption) can be used as a basis for welding thin nitinol wires.

PAW results showed that welding nitinol requires an increase in the level of atmospheric protection of the melting zone. Oxide phase formations have a cross-shaped form similar to titanium carbide, which was found in [11] when welding high-carbon nitinol. According to the authors of this work, phase formation occurred during the crystallisation of the weld. A high concentration of oxide phase formations can significantly affect the shape memory effect. The NiTi_2_O crystal lattice is incoherent with TiNi, which contributes to the retention of areas of mechanical stress during reverse deformation (shape recovery). An increase in the number of incoherent phases not only increases brittleness but also reduces the degree of shape recovery. An additional negative factor affecting the properties of the welded joint is a 1.5-fold increase in the concentration of the Ti_2_Ni phase. Due to the fact that the size of inclusions of this phase in the weld zone does not exceed 5 µm, and most of them are ~1 µm in size, they do not affect the macro ratio of nickel and titanium in the weld material. However, the brittleness of this phase, the detection of cracks in it [6], and the incoherence of the crystal lattice are negative factors that are likely to cause unsatisfactory results in mechanical tests of welded joints made by PAW. To eliminate these negative factors, it is advisable to increase the atmospheric protection of the melting zone during welding, as well as to select a heat treatment mode that provides for the decomposition of the Ti_2_Ni phase, whose melting point is significantly lower than that of TiNi. In this case, the heat treatment time should be sufficient to equalise the concentration heterogeneity. It should be noted that with TIG welding, the degree of oxidation of the weld metal is lower. This may be due to the larger volume of the protection zone and the greater laminarity of the protective gas flow when using a gas lens with a diameter of 19.5 mm. In PAW, despite the separate supply of plasma-forming and shielding gases, the compression of the electric arc in the opening of a small-diameter plasma-forming nozzle leads to an acceleration of the plasma flow. The high-speed plasma flow contributes to an increase in the intensity of mixing of the gas phase of the protective environment and the suction of air.

The CDW results showed that the type of welded joint of nitinol wires can significantly affect the prospects for the use of such welding. When welding wires with a diameter of 0.8 mm in the weld area, macro-cracks measuring 150–250 µm were found, as well as the presence of copper electrode material on the surface of the joint, which, along with unsatisfactory mechanical test results, raises doubts about the prospects for using this type of welding. At the same time, CDW is recommended in [17].

With CDW of 0.8 mm diameter nitinol wires in a 0.8 mm diameter hole in a 2.4 mm wire, a welded seam was obtained, which does not differ from the wire material over 60% of the area of the joined surfaces. No excess phases were found in the area of the changed structure, except for eutectic grains of TiNi + TiNi_3._ No changes in the number and size of Ti_2_Ni inclusions were observed. It should be noted that the presence of TiNi_3_ intermetallic compounds could lead to increased brittleness of the welded joint. Its presence in the eutectic composition probably reduces this negative effect on mechanical properties and allows achieving mechanical test results that make this type of welding promising for further development of recommended thermal cycles for obtaining high-quality welded joints of thin Nitinol wires.

One of the main difficulties in selecting thermal cycles for obtaining high-quality welded joints of thin nitinol wires, which usually include heat treatment at a temperature of 450–500 °C, is the limitation on such treatment of the entire product. Understanding the negative factors that affect the performance characteristics of a welded joint will allow us to rationally determine the types of treatment of the welded joint to improve these characteristics.

## 7. Conclusions

1. TIG welding under the recommended mode parameters, with sufficient argon protection of the melting zone of thin nitinol wire from atmospheric influences, can ensure the formation of a welded joint structure close to that of the base metal. The ability of the samples to restore their shape due to the superelasticity of the welded joint material is preserved.
2. PAW under the recommended conditions leads to the formation of NiTi_2_O oxides in the weld zone and an increase in the concentration of Ti_2_Ni intermetallics, which causes brittle fracture and negatively affects the ability to restore the shape of the welded joint of thin nitinol wire without additional thermal exposure.
3. CDW is promising for making welded joints of thin nitinol wires with close contact of the welded surfaces. The recommended CDW mode leads to the formation of eutectic-type TiNi + TiNi_3_ grains in the welded material. The thermal cycle for obtaining a CDW welded joint of thin Nitinol wires needs to be refined in order to increase the strength and improve the shape recovery of the welded joint.

## Figures and Tables

**Table 1:** Chemical composition of nitinol alloy wires.

Material	Chemical Composition, wt.%								
	Ni	C	Co	Cr	Fe	Nb	Ti	Cl + Br	Other
Wire with a diameter of 0.1 mm to 3.0 mm, according to ASTM F2063	54.5–57.0	≤0.05	≤0.05	0.15–0.3	≤0.05	≤0.025	rest	≤0.15	O ≤ 0.05; H ≤ 0.005; N ≤ 0.009
Wire, according to certificate	55.65	≤0.05	≤0.01	0.25	≤0.01	≤0.005	rest	≤0.01	O ≤ 0.05; H ≤ 0.002; N ≤ 0.003
Wire, according to micro-X-ray spectral analysis	55.45–56.03	≤0.05	≤0.01	0.25	≤0.01	≤0.005	44.55–43.97	≤0.01	O ≤ 0.05; H ≤ 0.002; N ≤ 0.003

**Table 2:** Recommended parameters for welding with a non-consumable electrode in an inert gas environment and microplasma welding.

Method Welding	Current, A	Voltage, V	Nozzle Diameter, mm	Shielding Gas Consumption, L·min^−1^	Plasma-Forming Gas Consumption, L·min^−1^	Heating Time, s
TIG-1	23	13	19.5 with gas lens	10	-	1
TIG-2	21	14	19.5 with gas lens	13	-	1
Microplasma	18	14	2.5	7	5	1

**Table 3:** Technical characteristics of the TKM-15U4 spot capacitor discharge welding machine.

Parameter Name	Parameter Value
Mains voltage, V	220
Mains frequency, Hz	50
Permissible voltage deviations, %	±5
Average power consumption, W	500
Maximum stored energy, J not less than	600
Working stroke of the electrode, mm	15 (+1.5; −0.5)
Useful electrode projection, mm	150
Compression force adjustment range, N	9.8–58.8
Capacity adjustment range, μF	100–1200 (step 100)
Capacitor charging voltage adjustment range, V	100–980

**Table 4:** Recommended parameters for resistance spot capacitor discharge welding.

Welded Joint	Capacitor Bank Capacity C_p_, μF	Capacitor Charging Voltage U_C_, V	Number of Choke Windings W_dr_, pcs	Electrode Compression Force Re, N
Ni-Ti, Ø 0.8 mm + Ni-Ti, Ø 0.8 mm	600–800	600	80	29,4
Ni-Ti, Ø 0.8 mm + Ni-Ti, Ø 2.4 mm	600–1000	600	80	29,4

**Table 5:** Chemical composition of the surface zone of the welded joint of the sample made by TIG welding.

Element	At. No	Netto	Mass, %	Mass Norm, %	Atom, %	Abs. Error, % (1 Sigma)	Rel. Error, % (1 Sigma)
O	8	427	5.22	5.43	15.39	1.67	32.02
Ti	22	57,746	63.67	66.32	62.79	1.82	2.85
Ni	28	11,020	27.12	28.25	21.82	0.82	3.02
Sum			96.01	100.00	100.00	-	-

## Data Availability

The original contributions presented in this study are included in the article. Further inquiries can be directed to the corresponding author.
